# Generation of AAVS1 integrated doxycycline-inducible CRISPR-Prime Editor human induced pluripotent stem cell line

**DOI:** 10.1016/j.scr.2021.102610

**Published:** 2021-11-24

**Authors:** Nike Bharucha, Jennifer Arthur Ataam, Alexandra A. Gavidia, Ioannis Karakikes

**Affiliations:** Department of Cardiothoracic Surgery and Stanford Cardiovascular Institute, Stanford University School of Medicine, Stanford, CA, USA

## Abstract

Prime editing uses the Cas9 nickase fused to a reverse transcriptase to copy a DNA sequence into a specific locus from a ‘prime editing’ guide RNA (pegRNA), eliminating the need for double-stranded DNA breaks and donor DNA templates. To facilitate prime editing in human induced pluripotent stem cells (iPSCs), we integrated a doxycycline-inducible Prime Editor protein (PE2) into the AAVS1 genomic safe harbor locus. Prime editing of iPSCs resulted in precise insertion of three nucleotides in HEK3 locus with high efficiency, demonstrating the utility of this approach. This engineered cell line can be used to edit a single or multiple genomic loci by introducing a target-specific pegRNA for precise and effective genome editing to facilitate disease modeling and functional genetics studies.

## 1. Resource table

**Table:** 

Unique stem cell line identifier	SCVIi028-A-1
Alternative name(s) of stem cell line	SCVi-15S1-PE2
Institution	Stanford University School of Medicine
Contact information of the reported cell line distributor	Ioannis Karakikes, ioannis1@stanford.edu
Type of cell line	iPSC
Origin	Human
Additional origin info	Age: 21
	Sex: Male
	Ethnicity: Not Hispanic or Latino
Cell Source	PBMCs
Method of reprogramming	SendaI Virus (CytoTune™-iPS 2.0 Sendai Reprogramming Kit)
Clonality	Clonal (Isolated using the Isocell supplied by iotaSciences to ensure mono-clonality)
Evidence of the reprogramming transgene loss (including genomic copy if applicable)	PCR, Western Blot, qPCR for sendai virus - negative
Cell culture system used	Matrigel-coated feeder-free culture, StemMACS iPS-Brew XF media
Type of Genetic Modification	Transgene generation
Associated disease	N/A
Gene/locus	AAVS1 (OMIM 102699)/ 19q13
Method of modification/site-specific nuclease used	CRISPR/Cas9
Site-specific nuclease (SSN) delivery method	RNP
All genetic material introduced into the cells	sgRNA for AAVS1 locus, pAAVS1-PE2-P2A-BFP donor plasmid
Analysis of the nuclease-targeted allele status	PCR for WT allele and Sanger sequencing and confirmation of integration by junction PCR
Method of the off-target nuclease activity surveillance	In silico prediction and Targeted PCR with Sanger sequencing
Name of transgene	PE2-P2A-BFP [Cas9(H840A)MMLV RT-P2A-BFP]
Eukaryotic selective agent resistance (including inducible/gene expressing cell-specific)	Positive (neomycin, and Dox-inducible puromycin)
Inducible/constitutive system details	TET-On
Date archived/stock date	07/2021
Cell line repository/bank	https://hpscreg.eu/cell-line/SCVIi028-A-1
Ethical/GMO work approvals	Patient was enrolled in the study by informed consent approved by the Stanford Institutional Review Board (IRB # 29904)
Addgene/public access repository recombinant DNA sources’ disclaimers (if applicable)	pAAVS1-NDi-CRISPRi (Gen1) was a gift from Bruce Conklin (Addgene plasmid # 73497 ; http://n2t.net/addgene:73497 ; RRID:Addgene_73497) pU6-Sp-pegRNA-HEK3_CTT_ins was a gift from David Liu (Addgene plasmid # 132778 ; http://n2t.net/addgene:132778 ; RRID:Addgene_132778) pTRE3G-PE2-P2A-BFP was a gift from Jesse Engreitz and Glen Munson.

## 2. Manuscript section expected contents clarification

### 2.1. Resource utility

This iPSC line can be used to edit any locus in the human genome using prime editing (PE) by transfecting a locus-specific prime-editing-guide-RNA and inducing the expression of the prime editor with doxycycline. This will be applicable for functional genetics studies like validating GWAS hits and disease modeling, as well as inserting tags/epitopes precisely into loci.

## 3. Resource details

The CRISPR-Cas system has revolutionized genome editing in the last decade due to its relative ease of use, lower cost and high programmability, as compared to other genetic engineering tools. There are now four classes of CRISPR-Cas tools available - nucleases, base editors, transposases and prime editors, of which, prime editors are the most versatile (Anzalone et al, 2020). Prime editors allow for precise edits of point mutations, all twelve possible base-to-base conversions, small insertions/deletions with fewer undesired mutations and with higher or similar efficiency than homology-directed repair, without double strand breaks or donor DNA. The prime editor protein (PE2) is a fusion between the *S.pyogenes* Cas9(H840A) nickase and an engineered Moloney Murine Leukemia Virus (MMLV) reverse transcriptase domain (Anzalone et al, 2019). This fusion protein can be directed to the desired locus by an engineered prime editing guide RNA (pegRNA) which, includes the target site in its spacer sequence and the desired edit in an extension at the 3′ end of the pegRNA. Once the target DNA is nicked, its 3′ end hybridizes to the primer binding site and using the pegRNA template, PE2 reverse transcribes the DNA with the desired edit.

**Fig. 1.**
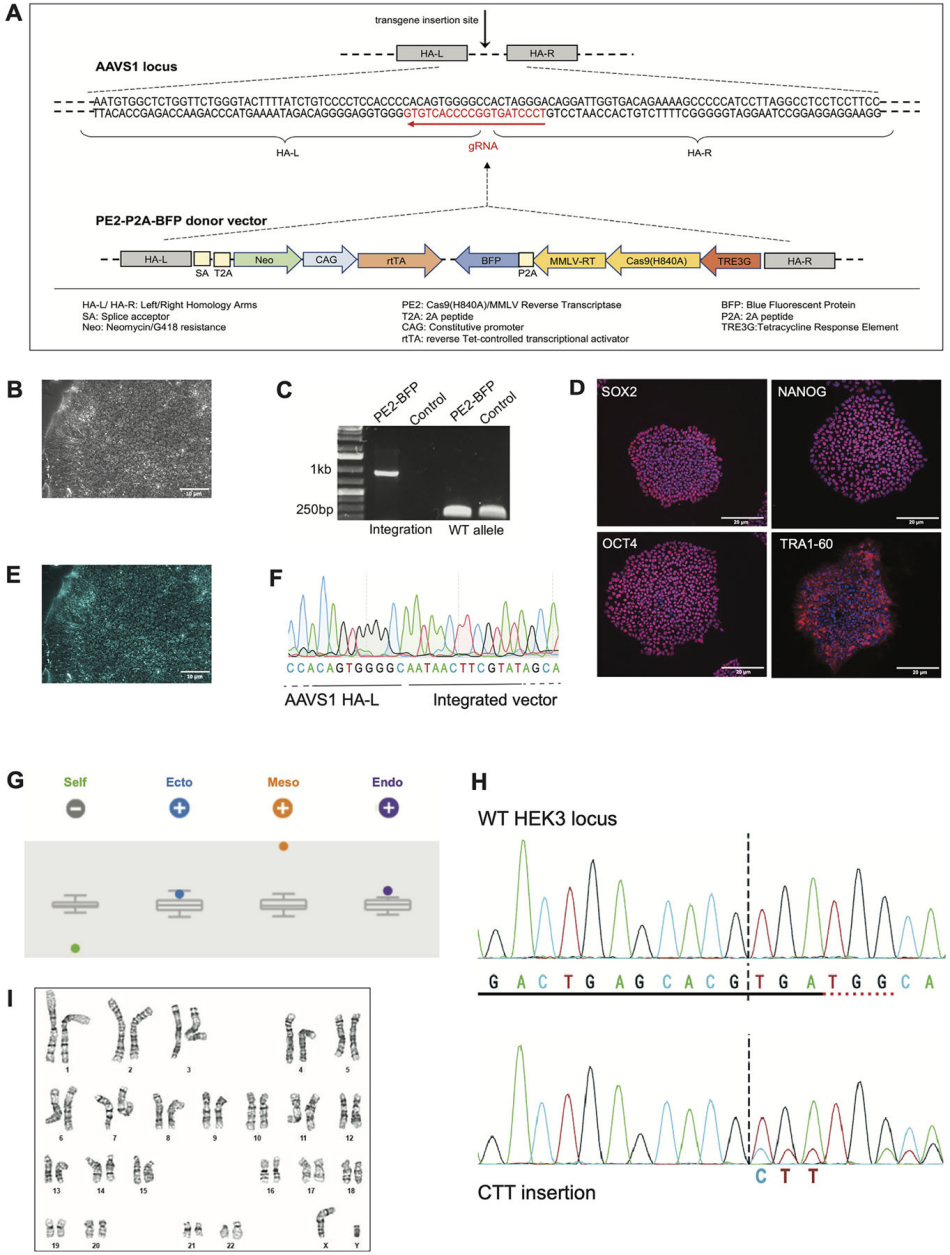
STR analysis karyotyping.

We generated a human iPSC line that inducibly expresses the PE2 protein that enables gene editing at potentially any locus in the genome by transfecting a pegRNA specific for the desired target locus. (See Tables 1 and 2). This line can be used for functional genomics studies, such as validating GWAS hits and inserting epitope tags or introducing SNPs and then differentiating the line into the cell-type of interest. The prime editor was integrated at the AAVS1 ‘genomic safe harbor’ locus within the PP1R12C gene (Mandegar et al, 2016). Transgenes integrated at this locus retain their transcriptional activity both in iPSCs, and upon differentiation into other cell types. The construct was designed such that PE2 is under transcriptional control of the Tetracycline regulatable promoter and can be activated by the addition of doxycycline when required, remaining transcriptionally inactive upon doxycycline withdrawal (Fig. 1A). Additionally, the PE2 is fused to Blue Fluorescent Protein (BFP) with a P2A peptide sequence, allowing for visualization of cells that are actively transcribing the PE2-P2A-BFP (Fig. 1E). After selecting for the integrated plasmid with the antibiotic G418, iPSC clones were selected and expanded for further characterization . The PE2-P2A-BFP-integrated cell line showed normal morphology (Fig. 1B). The correct insertion of the transgene at a single allele of the AAVS1 locus was verified by PCR amplification of the 5′ integration junction (1 kb). A different set of primers amplified across the cut site (250 bp) showed the intact WT allele (Fig. 1C). Pluripotency was verified by immunostaining for OCT3/4, SOX2, NANOG, TRA1-60 (Fig. 1D), and trilineage potential was confirmed by Scorecard at passage 35 (Fig. 1G). The cells showed normal karyotype at passage 35 (Fig. 1I). We validated the editing capability and utility of the PE2 engineered cell line by transfecting a pegRNA carrying a 3 bp (CTT) insertion targeting the HEK3 locus, followed by Sanger sequencing of the PCR-amplified DNA collected from the edited cells (Fig. 1H).

## 4. Materials and methods

### 4.1. Generation and maintenance of the iPSC line

The iPSCs were cultured in StemMACS iPS-Brew XF (Miltenyi Biotec) on Matrigel (BD Biosciences) coated plates at 37 °C and 5%CO2/5%O2 as described (Feyen et al, 2021). For transgene insertion, 250,000 iPSCs were nucleofected (1200 V, 20 ms, 1 pulse) with 60 pmoles sgRNA (Synthego) targeting the AAVS1 locus, 20 pmoles SpCas9 nuclease (Synthego) and 1 μg PE2-P2A-BFP plasmid using the Neon Transfection System (ThermoFisher Scientific) per the manufacturer’s instructions. When cells reached 75% confluency, they were dissociated by DPBS-EDTA at 37 °C for 7–10 min and replated in StemMACS iPS-Brew XF containing 5 μM Y-27632 (Selleckchem). For selection, the iPSCs were grown in the presence of 50 μg/ml G418 for 5 days. To ensure monoclonality, single-cell cloning was undertaken using the isoCell supplied by iotaSciences.f Expression of the transgene was confirmed by addition of 1.5 μg/ml Doxycycline Hyclate (Calbiochem) for 48 h.

### 4.2. Molecular cloning

The PE2-P2A-BFP fusion was PCR amplified from pTRE3G-PE2-P2A-BFP and cloned into NotI/AflII-digested pAAVS1-NDi-CRISPRi (Addgene#73497) using the In-Fusion HD cloning kit (Takara), replacing the KRAB-dCas9-P2A-mCherry cassette. The tetracycline-inducible vector contains the reverse tetracycline-controlled transcriptional activator (rtTA) as well as the tetracycline-response element (TRE3G). The rtTA is transcribed by a strong constitutive promoter (CAG) oriented in the opposite direction of the TRE3G element, which ensures no expression of the transgene can occur without addition of doxycycline. The vector contains left and right homology arms (HA-L/HA-R) that flank the genomic-cut site in the AAVS1 locus. A splice-acceptor (SA) site and a 2A peptide sequence (T2A) downstream of the HA-L arm allows for endogenous expression of a promoterless-Neomycin gene that confers resistance to Neomycin/G418.

### 4.3. PCR and sequencing

Genomic DNA was extracted using Quick Extract solution (Lucigen) and PCR-amplified with GoTaq HotStart polymerase (Promega). Integration of the pAAVS1-PE2-P2A-BFP vector at the AAVS1 locus was confirmed with vector-specific (within SA site) and AAVS1 locus-specific primers that amplified the 5′ integration junction (1 kb product). A second primer set (within HA-L and HA-R) amplified the WT AAVS1 junction spanning the cut site, which indicated presence of the WT allele (250 bp product). PCR cycling condition: 95 °C 2 min; 95 °C 15sec, 60 °C 15sec, 72 °C 1 min (40 cycles); 72 °C 1 min.

### 4.4. Immunostaining

The cells were fixed with 4% PFA for 10 min at 37 °C and washed 3 times for 5 min each with DPBS. They were then permeabilized in DPBS with 0.1% Triton for 10 min at room temperature, followed by blocking for 1 h at room temperature in DPBS/0.1% Triton X/1% BSA. Cells were incubated with primary antibodies at 4 °C overnight. The cells were then washed 3 times for 5 min each with DPBS and incubated with secondary antibody for 1 h at room temperature. After washing 3 times for 5 min each, a drop of NucBlue was added to counterstain the DNA.

### 4.5. Validation of the line for genome editing

The plasmid pU6-Sp-pegRNA-HEK3-CTT-ins (Addgene# 132778) expressing a pegRNA specific for a 3 bp insertion (CTT) of the HEK3 locus was electroporated into the PE2-BFP iPSCs. At 48 h post-electroporation, the cells were induced with 1.5 μg/ml Doxycycline. DNA was collected 72 h post-Doxycyclin induction and PCR-amplified, followed by Sanger sequencing to confirm target insertion.

## Figures and Tables

**Table 1:** Characterization and validation.

Classification	Test	Result	Data
Morphology	Photography	Typical human pluripotent stem cell morphology	Fig. 1B
Pluripotency status evidence for the described cell line	Qualitative analysis (Immunocytochemistry)	Positive for pluripotency markers: OCT3/4, NANOG, TRA1-60, SOX2	Fig. 1D
Karyotype	Karyotype (Wicell)	Normal male karyotype (46, XY), no clonal abnormalities detectedResolution: 425–450 bands	Fig. 1I
Genotyping for the desired genomic alteration/allelic status of the gene of interest	PCR across the edited site or targeted allele-specific PCR	PCR across integration site in edited and wild-type alleles.	Fig. 1C
	Transgene-specific PCR	N/A	N/A
Verification of the absence of random plasmid integration events	PCR/Southern	N/A	N/A
Parental and modified cell line genetic identity evidence	STR analysis (Wicell)	DNA Profiling 15 loci analyzed; matched to parental line.	Supplementary file submitted in the archive with journal
Mutagenesis/genetic modification outcome analysis	Sequencing (genomic DNA PCR or RT-PCR product)	Verified presence of integration in single edited allele and absence of integration in wild-type allele. Monoallelic transgene insertion.	Fig. 1F
	PCR-based analyses	Detection of correctly-targeted construct	Fig. 1C (Sample Lane 1)
	Southern Blot or WGS; western blotting (for knock-outs, KOs)	N/A	N/A
Off-target nuclease analysis-	PCR across top predicted likely off-target sites	PCR across predicted off-target site; Sanger sequencing	No off-target effect observed
Specific pathogen-free status	Mycoplasma testing by MycoAlert Detection Kit; passage 35	Negative	N/A
Multilineage differentiation potential	Spontaneous Differentiation; RNA isolation RNeasy kit (Qiagen); Taqman Scorecard (ThermoFisher Scientific)	Tri-lineage differentiation potential	Fig. 1G
Donor screening (OPTIONAL)	HIV 1 + 2 Hepatitis B, Hepatitis C	N/A	N/A
*Genotype - additional histocompatibility info (OPTIONAL)*	Blood group genotyping	N/A	N/A
	HLA tissue typing	N/A	N/A

**Table 2:** Reagents details.

Antibodies and stains used for immunocytochemistry/flow-cytometry			
	Antibody	Dilution	Company Cat # and RRID
Pluripotency Markers	Mouse anti-OCT3/4, mouse anti-TRA-1–60, rabbit anti-NANOG, mouse anti-SOX2	1:200	Santa Cruz Cat #SC-5279, Millipore Cat #MAB4360, Santa Cruz Cat #SC-33759, Cell Signalling Cat #4900S
Differentiation markers	N/A	N/A	N/A
Secondary antibodies	Goat Anti-Mouse IgG Alexa fluor 594, Goat Anti-Rabbit IgG Alexa fluor 488	1:800, 1:400	Invitrogen Cat #A11032, Invitrogen Cat #A11070
Nuclear stain	DAPI	1 drop	Invitrogen Cat #R37606
Site-specific nuclease			
Nuclease information	SpCas9	Synthego	
Delivery method	Nucleofection	Neon Transfection System (ThermoFisher)	
Selection/enrichment strategy	50 μg/ml G418		
Primers and Oligonucleotides used in this study			
	Target	Forward/Reverse primer (5′-3′)	
In Fusion Cloning	For cloning PE2-P2A-BFP into AAVS1-NDi-CRISPRi (Gen1)	Fw:CACTTCCTACCCTCGTAAACTTAAGGCCACCATGAAACGGACAG Rv:TGGGGTGGGCGATCGATTGCGGCCGCTTAATTAAGCTTGTGCCCCAG	
gRNA sequence	AAVS1 locus		
Junction PCR	To confirm integration at AAVS1 locus	Fw: TTGAGCTCTACTGGCTTCTGCGC Rv: GCCCTGTGGGAGGAAGAGAAGAGG (1 kb amplicon)	
PCR of WT allele	AAVS1 locus	Fw: CGGTTAATGTGGCTCTGGTT Rv: AGGATCCTCTCTGGCTCCAT (250 bp amplicon)	
PCR of HEK3 locus	HEK3 locus	Fw: ATGTGGGCTGCCTAGAAAGG Rv: CCCAGCCAAACTTGTCAACC	
Top off-target mutagenesis predicted site sequencing (identified using COSMID) (Cradick et al., 2014)	Chr22:48335634–48335655	Fw: GGAGAGGAGAAGAGGATACAGAC Rv: TCCAGAAGCCTGCAGGCTGA	
